# A Systematic Approach to Screen, Identify, and Correct Malfunctioning Interruptive Alerts

**DOI:** 10.1055/a-2646-6297

**Published:** 2025-08-20

**Authors:** EzzAddin Al Wahsh, Justin Juskewitch, Carol Eichenlaub, Courtney Holbrook, Pedro J. Caraballo

**Affiliations:** 1 AL Clinic, Minneapolis, Minnesota, United States; 2 Department of Laboratory Medicine and Pathology, Mayo Clinic, Rochester, Minnesota, United States; 3 Department of Information Technology, Mayo Clinic, Rochester, Minnesota, United States; 4 Department of Medicine, Mayo Clinic, Rochester, Minnesota, United States; 5 Department of Quantitative Health Sciences, Mayo Clinic, Rochester, Minnesota, United States

**Keywords:** decision support systems, clinical, alert fatigue, clinical alerts, alert malfunctions, interruptive alerts

## Abstract

**Background:**

Interruptive alerts can negatively impact clinical workflows and contribute to alert fatigue, provider frustration, and burnout. Given that interruptive alert overriding is a heterogeneous and recurring phenomenon, occurring across different organizational contexts with varying characteristics and circumstances, we hypothesize a pragmatic approach with multimodal interventions to address malfunctioning alert populations and maintain those contributing to better patient care.

**Objective:**

This study aimed to develop a systematic approach to screen, identify, and correct malfunctioning interruptive alerts within a tertiary healthcare system.

**Methods:**

We performed screening by assessing the alert population, exploring available resources, and defining alert population inclusion and exclusion criteria. We identified interruptive alerts and then conducted an exploratory analysis. We shared insights from discussions with our expert panel to validate our findings and find gaps in current alert monitoring. We then performed focus groups and interviews as part of a root cause analysis. To address the findings of these investigations, we prioritized which alerts to improve, evaluated solutions, and recommended steps to improve our governance structure.

**Results:**

We developed an approach to assess around 1,500 unique alerts in a tertiary center from January to June 2023. We introduced two approaches to visually analyze alert populations: alert-focused analysis and people- and systems-focused analysis. We utilized an expert panel to further enhance the power and speed of alert evaluation and then investigated one emerging alert with focus groups, identifying root causes for its malfunction. This alert demonstrated how enterprise practice changes, coupled with design and cultural issues, can trigger significant alert malfunctions.

**Conclusion:**

A multi-modal intervention approach is needed to evaluate interruptive alerts and act quickly on findings. Utilizing both analytical and nonanalytical methods can work in synergy to facilitate this framework. Such approaches may reduce time and be valuable tools for optimally allocating resources to tackle institutional alert challenges.

## Background and Significance

Interruptive alerts implemented in the electronic medical record (EMR) have the potential to improve patient care. However, such alerts can also negatively impact clinical workflows and contribute to alert fatigue, provider frustration, and burnout. 1 2 3 4 5 6 7 Alert malfunction refers to an undesired output of a given alert within the EMR environment during a given clinical workflow. 8 An alert can malfunction due to inadequate alert logic, incorrect knowledge content, user interface issues, or conflicting alert dependencies, such as the same trigger conditions or overlapping workflows.

Changes in clinical recommendations, workflows, information technology releases, or institutional policy can all contribute to alert malfunction and resulting overrides. As such, similar alert malfunction patterns tend to occur across healthcare organizations and clinical tasks. 9 10 11 12 13 14

Due to the growing number and complexity of interruptive alerts (beyond computerized physician order entry [CPOE] flagging drug–drug and drug–allergy interactions), organizations now work to properly govern and maintain the alert population that is contributing to better patient care. 15 16 17 Multiple approaches to managing interruptive alerts have been attempted. 3 11 18 Some methods focus on individual alerts but have encountered challenges within complex clinical environments. Tertiary medical care systems have leveraged external tools to help maintain interruptive alerts. 10 18 Iterative data-driven (analytical) improvements to alert malfunction have also proven valuable. 19 Current evidence supports integrating multiple interventions into a combined single strategy to effectively improve real-world alert system applications. 20

The complexity of clinical workflows leads to an increased need for clinical decision support (CDS) interventions within an integrated, interruptive alert optimization program. 11 Our challenge was to develop a practical framework to evaluate many interruptive alert interactions following a standardized and reproducible approach using available internal tools and existing governance infrastructure.

## Objectives

This study aimed to develop a systematic approach to screen, identify, and correct malfunctioning interruptive alerts within a healthcare system and demonstrate its utility.

## Methods

This study was executed using data from Mayo Clinic's integrated EMR (Epic, Verona, Wisconsin, United States), whose hospital system is based across multiple geographic locations, including Rochester, Minnesota, United States; Jacksonville, Florida, United States; Phoenix, Arizona, United States; and its community health system sites across southeast Minnesota, United States and northwest Wisconsin, United States. The team consisted of a clinical informatics physician, clinical informatics nurse, EMR analyst, enterprise IT application analyst, and clinical informatics fellow. The team met regularly every 2 weeks to refine the framework methodology and apply it for alert analysis.

We utilized available EMR tools such as SlicerDicer and User Web (Epic, Verona, Wisconsin, United States). This work focused on interruptive alerts in the inpatient setting that were overridden (not accepted or acknowledged by the end user) when they were displayed in the clinical workflow.

### Screen

**Fig. 1:**
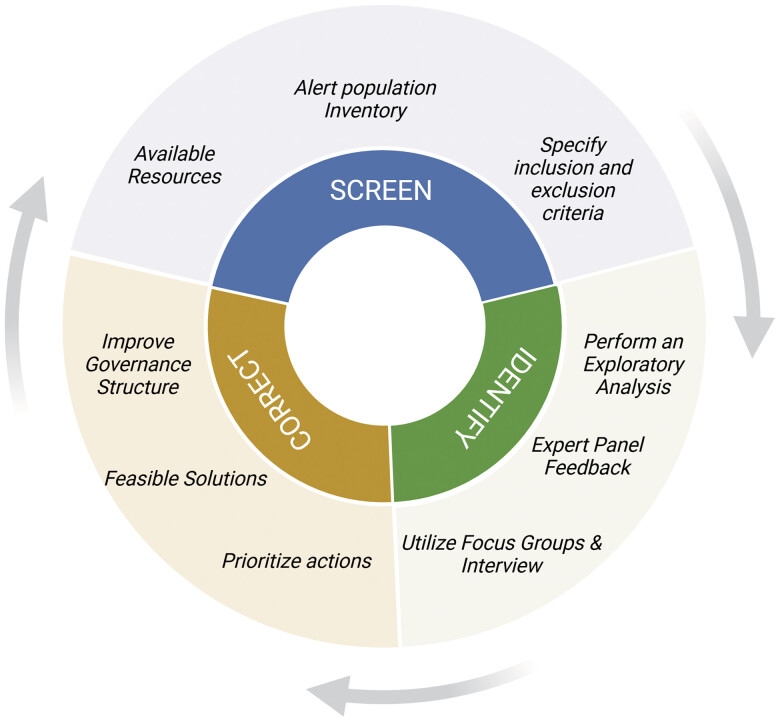
A framework to approach the interruptive alerts population: screen, identify, and correct.

In this phase, we performed screening by exploring available resources, assessing the alert population, and defining our alert population inclusion and exclusion criteria ( Fig. 1 ).

#### Available Resources

We surveyed both institutional human and nonhuman resources available for the alert screening framework. We also identified key individuals within the CDS governance structure (institutional CDS subcommittee) to provide leadership support for using these resources (e.g., participation in operational meetings, assistance in forming focus groups, and granting access to reporting tools and data models to analyze alert trends).

#### Alert Population Inventory

We needed to assess which unique alerts exist in our EMR and what kinds of alerts they are. Additionally, we needed to identify any blind spots and determine if other systems outside of the EHR have interruptive alerts that could impact the hospital environment (e.g., laboratory information system, radiology information system, and pharmacy information system).

#### Inclusion and Exclusion Criteria

We defined our alert population of interest as all interruptive alerts generated from January to June 2023. The inclusion criteria for our analysis included encounters classified as “hospital encounters” and a display mode set to “interruptive.” We included all clinical users (physicians, nurses, pharmacists, etc.) and alert records. We excluded alerts that had “any action taken” and those related to CPOE drug–drug and drug–allergy interactions.

### Identify

In this phase, we conducted an exploratory analysis and validated our findings with an expert panel. We also interviewed focus groups to explore gaps, particularly for alerts that the expert panel was unfamiliar with ( Fig. 1 ).

#### Exploratory Analysis (Detailed Process)

We utilized the alert data model and interactive dashboard in SlicerDicer, a self-service reporting tool within our institutional EMR. To visualize alert trends, we set the population base to “all our practice advisories” with the following criteria: “not any action taken?,” “encounter type: hospital encounter,” and “display mode: interruptive.” We sliced by “our practice advisory,” defaulted measures to count, filtered alerts based on the most firing events. Then, sorted findings were sorted sequentially to consider potential contextual factors described below.

First method, we adopted a people- and systems-focused analysis (PSFA). We selected the encounter type (hospital), performed sequential sorting, and incorporated hospital location (since our institution has multiple locations) into our population definition. Then, we sorted by users and incorporated the top category (nursing) into our population characteristics. We followed a similar iterative framework for specific hospital locations and departments. After considering these four contextual factors, we sorted for the top-firing alerts.

Another method, the alert-focused analysis (AFA) approach, which did not consider contextual factors and only sorted the top interruptive alerts. The AFA showed top alerts regarding total firings and the rate of change in firings per day over the study period. An inflection point was defined as the start of a change in the slope of the trend line between two time points, where the frequency at least doubles in either direction. Table 1 summarizes the key differences between the PSFA and AFA approaches.

**Table 1:** Comparison between the AFA and PSFA approaches for alert analysis

Approach	Benefit	Drawback
Alert-focused analysis (AFA)	Fewer steps Easier to communicate. Does not require knowledge of workflows/practice	It does not consider contextual factors (site/practice) and change management
People and systems-focused analysis (PSFA)	Considers contextual factors (site/practice) Considers change management	More steps It may not show the impact of technology clearly

#### Expert Panel Feedback

The experts' panel meeting is an existing component of our institutional CDS governance structure. We then brought PSFA and AFA findings to an expert CDS panel comprising over thirty subject matter experts who meet bimonthly to discuss institutional CDS updates and trends. This expert panel included physicians, nurses, pharmacists, operational managers, EMR technical analysts, IT analysts, clinical informaticians, and CDS governance leadership.

The top overridden alerts and their trends were visually shared with the expert panel. The expert panel had real-time access to the alert name, number, and trend. The panel's coordinator asked the panel to share their thoughts on why the alert was needed, why it was overridden, and whether more work was required to improve it.

Table 2 displays the eight most malfunctioning alerts that resulted in the highest number of overrides in the alert population over a 6-month period. Table 3 summarizes the panelist reviews of the top overridden alerts.

**Table 2:** Interruptive alerts that contributed to the majority of firings (only stable alerts)

Interruptive alert name	Category	Total events	No action taken	Contribution to overall alert population overrides (%)	Override rate a (%)
Inpatient before admission, medications not verified	Safety	303,166	299,523	13	99
Inpatient nursing suicidal ideations	Safety	141,431	138,720	6	98
Emergency department is not on the treatment team	Quality of care	169,922	165,428	7	97
Inpatient nursing early screening for discharge	Compliance	120,552	103,807	4	86
Incomplete blood documentation	Compliance	117,186	112,975	5	96
Inpatient nursing discharge planning assessment	Compliance	54,364	46,663	2	86
Inpatient allergies not verified	Meaningful use	101,397	99,166	4	98
Inpatient nursing admission nutrition screen/consult	Compliance	99,339	85,785	4	86

a Average override rate for the remainder of the population is 75%.

**Table 3:** Panelists' comments on overridden alerts

Alert name	Context and criteria	Main concern	Explanation
Inpatient prior to admission, medications not verified	To prompt providers to review and verify home medications while admitting patients to the hospital. Patient should have an active inpatient encounter, “prior to admission,” the medication list has not been verified	Design	The medication reconciliation process involves both pharmacists and physicians. Alerts fire if physicians mistakenly enter the process before pharmacists complete their part
Inpatient nursing suicidal ideation	For inpatient nursing staff to identify patients with suicidal ideations, to place a suicide assessment consult. Triggered after completion of the suicide assessment by nursing staff, if the age of the patient, location, and active discharge order fulfill the criteria	Design	Alerts are triggered based on a diagnosis, such as depression, from the problem list rather than the admission diagnosis, which causes misalignment with workflows and lacks action within the alert
Emergency department is not on the treatment team	To prompt providers to review and verify home medications during hospital admission. The patient must have an active inpatient encounter, and the “prior to admission” medication list must not have been verified	Design	Alerts accidentally fire when an outpatient provider accesses a patient's record. Override rates depend on patient volume and acuity
Inpatient nursing early screening for discharge	To support nursing staff in initiating early discharge planning by prompting a care management consult only when documentation indicates a need	Regulatory	The practice requires an alert to interrupt the user before the discharge process to ensure compliance
Inpatient nursing discharge planning assessment	To support nursing staff in initiating early discharge planning by prompting a care management consult only when documentation indicates a need. It ensures the timely involvement of care management for patients with discharge needs identified at the point of admission. Alert fires for patients who are currently admitted or have an order to inpatient or observation status, if nurses have already completed their assessment, fires to registered nurses, licensed practical nurses, and clinical nursing specialists. Excludes inpatient psychiatry units	Regulatory	Similar to the previous rule, alerts are required when discharging patients by the nursing team to meet the regulatory standard
Incomplete blood documentation	The alert is triggered when blood transfusion documentation remains incomplete after a designated period, supporting compliance with documentation standard and promoting patient safety. Triggering criteria include completion of a transfusion without corresponding documentation within a specified time window, the patient being flagged for long-term care or social services, and the current end user being a member of the care team. The alert only fires for inpatient encounters and is limited to registered nurses. Emergency Department encounters are excluded	Regulatory	Documentation requirements that cannot be completed during the encounter triggering an alert
Inpatient allergies not verified	This alert prompts verification of patient allergy documentation if it has not yet been reviewed during the current hospital encounter. It ensures up-to-date allergy records to support safe medication ordering and clinical decision-making. The alert is triggered for actively hospitalized patients when the end user initiates actions such as entering an order or opening an order set. Providers in emergency medicine are excluded from this alert	Workflow overlap	Similar to a medication reconciliation alert, a physician may encounter duplicate alerts if they access the allergy section before the nurse or pharmacist completes their part
Inpatient nursing admission nutrition screen consult	This alert is designed to initiate a dietitian consult when the modified malnutrition screening tool (MST) indicates that a patient is at nutritional risk. It supports early nutritional intervention based on nursing admission documentation and displays as a high-priority alert. The alert is triggered for patients with an active inpatient encounter, after nursing completes the nutrition assessment, and if the MST score meets the defined threshold	Regulatory	Regulatory rule

Table 2 displays the eight most malfunctioning alerts that resulted in the highest number of overrides in the alert population over a 6-month period. Table 3 summarizes the expert panel's reviews of these top overridden alerts and potential reasons for alert overrides. Expert panel concerns were categorized into three groups: design (e.g., conflicting interests among multiple end users). Regulatory Compliance (e.g., alert was designed as a reminder for legal or accreditation compliance issues). Workflow (e.g., alert was triggered at an inappropriate clinical workflow step).

#### Focus Groups and Interviews

Focus group interviews involved both technical and nontechnical groups. When evaluating a misfiring alert, the technical focus group in our example instance comprised a clinical informatics fellow, an informatics nurse specialist, an IT specialist, and an EMR technical analyst. This technical focus group reviewed the build of the alert, including triggers, frequency of firing, and end-user types.

The nontechnical focus group involved clinical proponents from the site identified with the highest overridden alerts, operational manager(s), and a clinical informatics fellow. This nontechnical focus group reviewed the clinical rationale behind this alert (e.g., Does it add value to the practice? Why do end users override the alert? Did the alert improve the practice toward the goal of the alert?). Both focus groups fulfilled the definition of Wright's taxonomy of CDS malfunction. 8 21

### Correct

In this phase, we prioritized alerts, evaluated feasible solutions, and recommended improvements to our governance structures ( Fig. 1 )

#### Prioritize Actions

Given the insights from earlier stages, we prioritized which alerts to address and provided recommendations to the CDS governance leadership regarding alert ownership at the practice level.

#### Feasible Solutions

We evaluated technical solutions for prioritized alerts based on feasibility, cost, and institutional policies. As such, optimal solutions often differed for each prioritized alert based on this analysis.

Technical solutions considered were based on the options available within our EMR, including alterations to triggering rules, workflow display modalities, and user type/location/service targeting.

#### Improve Governance Structure

We shared suggested improvements on the governance structure with our CDS subcommittee. At our organization, a CDS subcommittee functions as the governing body for new interruptive alert requests. Historically, new alert proponents submit follow-up reports within 3 months of implementation to determine their value and impact. The CDS subcommittee improved its alert governance by longitudinally incorporating dashboard and EMR reporting tools to assist with ongoing alert monitoring and evaluation.

## Results

We created an iterative framework to monitor a population of EMR alerts through screening, identifying, and correcting malfunctions within our institution's existing governance structure ( Fig. 1 ).

Using this framework, we surveyed the alert population from January to June of 2023. Our institution recorded a total of 51,132,636 alert events generated in the hospital setting. These alerts originated from approximately 3,125 unique alert base records. Among these unique alerts, nearly half (1,500) were classified as interruptive alerts. Overall, 92.7% of alert events were generated from passive or nondisplayed alerts. Interruptive alerts accounted for only 3,732,682 of the total alert events, with 72.5% of interruptions occurring in the hospital setting.

Among the 1,500 unique alert bases, eleven unique bases accounted for nearly 50% of all alert override events. Override rate (the total number of events not accepted or acknowledged divided by the total number of alerts presented to users) was trended over the study period. The top 8 (out of 11) alerts showed a stable trend and were therefore classified as “stable.” The remaining three alerts, which saw a sharp increase, were labeled as “emerging alerts.” The average override rates for stable alerts, emerging alerts, and the remaining unique alert population were 95.0, 95.3, and 74.9%, respectively.

Our visual analytical approaches, AFA and PSFA, identified the same interruptive alert trends (stable and changing). Table 1 shows how PSFA highlighted contextual factors (sites and roles) and served in the CDS governance strategy to evaluate malfunctioning alerts early on. The PSFA approach emphasized contextual elements such as encounter setting (hospital), user role (nurses), location (two hospitals), and department (Emergency Department).

Table 1 shows a comparison between the AFA approach and the PSFA approach. AFA was more accessible to apply and communicate with, and did not require knowledge of workflows. The most overriding impacts were stable (eight alerts, 40% of EMR overrides). This approach enabled the expert panel to focus on alerts with the most significant impact for an efficient review.

### A Showcase of a Comprehensive Approach

**Fig. 2:**
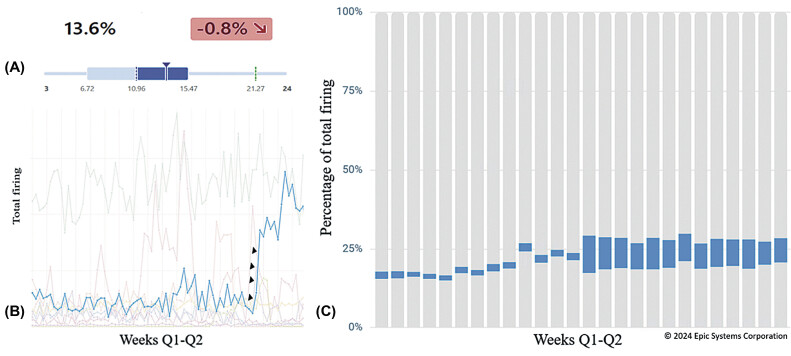
( **A** ) The dashboards for executives to monitor alert performance cannot answer the “why” behind changing trends. ( **B** ) Plotting trends of alerts' population showed changing trends (arrow heads). ( **C** ) The impact of one alert with a changing trend (blue) to the entire alert population around the time of ending a universal masking for COVID.

One of these three “emerging alerts” rose sharply in April 2023 (infection-isolation mismatch alert), with one location contributing to most of the overrides for this particular alert when compared with other sites. The infection-isolation mismatch alert ( Fig. 2 ) notifies end users that a particular patient is not receiving the appropriate isolation precautions for a given infection. Originally, this alert was designed to help infectious disease control administrators survey patients and remind providers to comply with guidelines. Later, the alert was modified to interrupt end users, nurses, and providers with institutional isolation guidelines.

The expert panel was unfamiliar with this alert, so technical and nontechnical focus groups were assembled to understand the background and motives behind this alert. The technical focus group discovered no logical errors or production changes over the study period. The nontechnical focus group interview revealed multiple factors that contributed to this override phenomenon.

Institutional factors identified impacting this alert included an environmental shift that occurred around the time of the spike, where the enterprise ended universal masking for COVID-19. The enterprise turned to a “protective environment” approach, requiring staff to universally mask only for high-risk patients, like those who are immunocompromised.

Design factors identified impacting this alert included the targeting of two distinct but discrepant user groups (infection control administrators and bedside nurses). Infectious disease proponents intended this alert to generate a surveillance list of isolation mismatches for infection control administrators to flag patients for awareness and potential intervention.

Cultural factors influenced the situation at this one site, as bedside nurses felt that the alert was outside their scope of practice, given the role of infection control practice surveillance. Many of these bedside nurses were new to this clinical role due to high staff turnover. Additionally, the complexity of the patients and infectious disease protocols at this one site was greater than the rest of the institution.

Our recommendation was to redesign this alert to consider a narrower end-user scope (only infection control administrators) with fewer constraints (silent alerts and change lock-time).

## Discussion

In a few months and existing CDS governance structure, this framework permitted the screening of a large population of EMR alerts and pragmatically identified and directed the correction of malfunctioning ones. We combined both technical and nontechnical approaches to complete an entire cycle of alert population monitoring (screen > identify > correct). In this iteration, we identified and selected an isolation infection mismatch alert due to its changing performance characteristics, identified the institutional, design, and cultural factors leading to this performance change, and implemented targeted improvements for this EMR alert.

This approach for evaluating interruptive alerts with excessive override patterns produced a targeted positive improvement in overall alert portfolio performance, utilizing the existing CDS governance structure and resources. Previous attempts to correct individual interruptive alerts will lose cost-effectiveness over time within a growing interruptive alert population. This framework utilized built-in analytical tools at no cost and leveraged our institution's existing CDS governance structure. 22

We provided a list of the most common interruptive alerts in a tertiary center with quick analyses using expert panel consensus. Compared with the quality improvement approach, 10 this approach helped us iterate and screen most interruptive alerts within one session. It aligned with quarterly governance cycles to evaluate interruptive alerts. Literature described the need for interruptive alert stewardship programs, especially regarding governance and design implementation/de-implementation. 23 Our work integrated an existing expert panel to be part of a stewardship effort in a functional and structured way.

AFA and PSFA are steps to standardize analytical approaches and documentation for interruptive alerts within an organization, regardless of the problem. The use of a single expert panel with a multidisciplinary technical and nontechnical membership helped avoid significant rework, benefiting from broad institutional subject matter expertise. While the medical literature suggests expert validation to be a robust yet resource-intensive detection method, we leveraged our expert panel to validate multiple alerts in one session visually, minimizing additional resource commitment. 24

Use of this framework also highlighted the significant regulatory component of our organization's alert burden. Around half of the interruptions were related to alerts with regulatory and compliance considerations. Such a high percentage is likely attributed to the nature of our matrix organization, which has multiple oversight structures that can have overlapped and even conflicting regulations. 16 Many interruptive alerts prompt users to act but allow them to bypass the alert, as they are intended to serve only an advisory role.

The Infection-Isolation mismatch alert also provides an excellent example of an emerging EMR alert that contributed to almost 10% of overridden alerts due to changes in COVID-19 precautions. Previous literature has also indicated similar malfunctions in alert systems related to COVID-19 precautions, emphasizing the need to involve stakeholders without pointing to changes in governance structures. 19

Key take-home lessons for this approach included the need for CDS champions from the practice to be part of interruptive alert monitoring and evaluation, the need for an ongoing operational owner of a specific alert after it was deployed into the practice, and the need for such an approach to be integrated into any existing institutional CDS governance structure, which is consistent with existing literature. 22 25

This framework can be generalized to institutions with similar alert data models and expanded to ambulatory and long-term facilities. One limitation is that we used our EMR vendor's existing alert data model and visualization tools for the AFA and PSFA analyses. Furthermore, the naming of alerts for specific tasks is not standardized across different EMRs or even organizations using the same EMR system, hindering interoperability. 11 26 27 Such inconsistency makes it challenging to share knowledge about alerts both between different organizations and even within the same EMR system across various institutions.

This framework was developed to assist institutions in appropriately allocating resources and improving governance for misfiring EMR alerts. More research is needed to explore the long-term impact of this approach on both alert fatigue and patient outcomes.

### Ethical Considerations

We maintained transparency by notifying the interviewees and focus groups, including panel experts, about the study's purpose. Recordings were made only after their voluntary consent was obtained, ensuring the study's ethical standards were upheld. Given the nature of interruptive alerts, the study design, and the cohort analytical tools used, this study was Institutional Review Board exempt, as we did not require protected health information.

## Conclusion

Multi-intervention approaches are needed to evaluate interruptive alerts and act quickly on findings. Utilizing both analytical and nonanalytical methods, this framework can reduce time and effectively allocate resources to tackle institutional alert challenges. Adopting such a framework may lead to more cost-effective use of alert systems and improved outcomes for patients and providers alike.

## Clinical Relevance Statement

Combining alert-focused and system-focused analyses significantly improved the efficiency of identifying and reviewing interruptive alerts. By developing a framework that included expert panel reviews, user focus groups, and a structured approach to revising alerts, we were able to standardize the review framework and enhance its efficiency.

## Multiple-Choice Questions

1. What human role did the study find essential to improving the governance structure and sustaining this framework to enhance interruptive alerts?
  - IT analyst
  - Clinical informatician
  - Project manager
  - CDS champion
  **Correct Answer:** The correct answer is option d. CDS champion. We noticed a gap in the governance structure when maintaining interruptive alerts. The CDS subcommittee will follow up after deploying an interruptive alert for 6 months. We noticed an uncertainty on who owns the framework of alert refining concerning usability and clinical outcomes from the clinical side. Given the highly changing clinical practice environment, a clinical liaison—a CDS champion—can support filling this gap in the governance structure and facilitating our iterative framework.
2. How did the expert panel add value to this framework?
  - It identified gaps in maintenance in a population of alerts in one session.
  - It provided us with the most accurate answers.
  - Clinical experts understood the reasons for overriding every alert.
  - It allowed for more time with each expert individually.
  **Correct Answer:** The correct answer is option a. It identified gaps in maintenance in a population of alerts in one session. The expert panel served as a central hub where we could validate our findings from the screening stage. We presented a group of panelists with our top overridden alerts within our population. The panel consisted of both clinical professionals and nonclinical operational and IT experts. While the experts agreed on the overall maintenance status of a particular alert, they could not provide precise details. The expert panel meetings' existing structure allowed us to validate all our top overridden alerts in one session, helping us focus on high-impact alerts.
